# Prevalence of cardiovascular disease among Asian, Pacific Islander and multi-race populations in Hawai’i and California

**DOI:** 10.1186/s12889-023-15795-5

**Published:** 2023-05-15

**Authors:** Beth Waitzfelder, Latha Palaniappan, Alexandra Varga, Timothy B. Frankland, Jiang Li, Yihe G. Daida, Joseph Keawe’aimoku Kaholokula, Adrian Matias Bacong, Andreea M. Rawlings, Sukyung Chung, Connor Howick, Stephen P. Fortmann

**Affiliations:** 1 Center for Integrated Health Care Research, Kaiser Permanente Hawai’i, Honolulu, HI USA; 2 Stanford University School of Medicine, Stanford, CA USA; 3 Kaiser Permanente Center for Health Research, 3800 N. Interstate Ave., Portland, OR 97227 USA; 4 Palo Alto Medical Foundation Research Institute, Center for Health Systems Research, Sutter Health, Palo Alto, CA USA; 5 Department of Native Hawaiian Health, John A. Burns School of Medicine, University of Hawai’i, Honolulu, USA; 6 Genesis Research, Hoboken, NJ USA; 7 Kaiser Permanente Bernard J. Tyson School of Medicine, Pasadena, USA

**Keywords:** Cardiovascular disease, Stroke, Asian, Pacific Islander, Filipino, Native Hawaiian

## Abstract

**Background:**

Cardiovascular disease (CVD) remains the leading cause of death in the US. CVD incidence is influenced by many demographic, clinical, cultural, and psychosocial factors, including race and ethnicity. Despite recent research, there remain limitations on understanding CVD health among Asians and Pacific Islanders (APIs), particularly some subgroups and multi-racial populations. Combining diverse API populations into one study group and difficulties in defining API subpopulations and multi-race individuals have hampered efforts to identify and address health disparities in these growing populations.

**Methods:**

The study cohort was comprised of all adult patients at Kaiser Permanente Hawai’i and Palo Alto Medical Foundation in California during 2014–2018 (*n* = 684,363). EHR-recorded ICD-9 and ICD-10 diagnosis codes were used to indicate coronary heart disease (CHD), stroke, peripheral vascular disease (PVD), and overall CVD. Self-reported race and ethnicity data were used to construct 12 mutually exclusive single and multi-race groups, and a Non-Hispanic White (NHW) comparison group. Logistic regression models were used to derive prevalence estimates, odds ratios, and confidence intervals for the 12 race/ethnicity groups.

**Results:**

The prevalence of CHD and PVD varied 4-fold and stroke and overall CVD prevalence varied 3-fold across API subpopulations. Among Asians, the Filipino subgroup had the highest prevalence of all three CVD conditions and overall CVD. Chinese people had the lowest prevalence of CHD, PVD and overall CVD. In comparison to Native Hawaiians, Other Pacific Islanders had significantly higher prevalence of CHD. For the multi-race groups that included Native Hawaiians and Other Pacific Islanders, the prevalence of overall CVD was significantly higher than that for either single-race Native Hawaiians or Other Pacific Islanders. The multi-race Asian + White group had significantly higher overall CVD prevalence than both the NHW group and the highest Asian subgroup (Filipinos).

**Conclusions:**

Study findings revealed significant differences in overall CVD, CHD, stroke, and PVD among API subgroups. In addition to elevated risk among Filipino, Native Hawaiian, and Other Pacific Islander groups, the study identified particularly elevated risk among multi-race API groups. Differences in disease prevalence are likely mirrored in other cardiometabolic conditions, supporting the need to disaggregate API subgroups in health research.

**Supplementary Information:**

The online version contains supplementary material available at 10.1186/s12889-023-15795-5.

## Introduction

Cardiovascular disease (CVD) is the leading cause of death in the US, with annual costs estimated to be $363 billion in 2016–2017 [1]. The progression of CVD occurs over many years and is influenced by a series of demographic, clinical, cultural, and psychosocial factors that vary across populations. In the US, there have been a growing number of studies designed to better understand these factors among Asians and Pacific Islanders (APIs). From 2010 to 2020, the Asian population in the US (alone and in combination with other races) increased nearly 40%, and the Pacific Islander population (alone and in combination) increased nearly 30% [2]. Compared to 2010, the 2020 census also showed a dramatic, 2.75-fold increase in individuals reporting two or more races [3]. More than half of Native Hawaiians (56%) and Other Pacific Islanders reported more than one race in 2020, and Asians had the second highest rate of reporting more than one race (17%) [3]. Emerging research suggests that some Asian subgroups, and Pacific Islanders, may be at higher risk for CVD and poorer outcomes than NHW, and that there are important differences among API subgroups [4–8]. Considering the rapid growth of both single and multiple-race populations, their health will have an increasing impact on future US health care needs and costs.

Knowledge gaps about the health of single and multi-racial Asian and Pacific Islander populations have persisted in part due to the problematic practice of aggregating broad and diverse Asian and Pacific Islander populations for research purposes. This practice can result in masking the extent of health disparities across these populations, and limit efforts to identify underlying drivers of health inequity [9–11]. The US Census provides data for 17 Asian ethnic groups, the largest being Chinese, Asian Indian, and Filipino [12]. The Pacific Islander population is also comprised of diverse ethnic groups. As the largest Pacific Islander group, Native Hawaiians are an Indigenous US population with a distinct social and cultural history, including negative health impacts associated with colonization [11]. In addition to Native Hawaiians, the broader Pacific Islander race category includes smaller populations, such as Samoans, Tongans, Marshallese, and Fijians [12]. In contrast to Native Hawaiians, these Pacific Islander groups often experience immigration challenges when moving to the US as well as unique acculturative stressors (e.g., language barriers) when residing in the US [11].

Limited evidence suggests that there is up to a six-fold difference in CVD rates among API subpopulations. Previous studies have shown the prevalence of CVD among API subpopulations ranging from 1.7% to 5.2%; stroke from 0.3% to 1.8%; and peripheral vascular disease from 0.9% to 3.4% [9]. Recent studies have highlighted both the need to disaggregate API subgroups [7, 8, 13, 14] and relatively higher CVD burden among some subpopulations, particularly South Asians and Filipinos [6, 15, 16]. Limitations in the classification of API ethnic and multi-racial groups in health research has greatly hampered our ability to detect and reduce the burden of CVD among APIs and multi-race groups. The Cardiovascular Disease among Asians and Pacific Islanders study (CASPER) provides a unique opportunity to leverage electronic health record (EHR) data, including detailed self-reported race/ethnicity data, to characterize CVD prevalence among large, well-defined single race and multiple race API populations in California and Hawai’i.

## Methods

### Study sites

The study was conducted at Kaiser Permanente Hawai’i (KPHI) and Palo Alto Medical Foundation (PAMF) in California. California and Hawai’i have the largest resident populations of Asians and Pacific Islanders in the US, with 52% of the nation’s Pacific Islanders and 37% of Asian Americans [2]. Hawai’i has the highest percentage of people reporting two or more races in the US 2020 Census (25%) [2]; California is fifth with nearly 15%. In the San Francisco Bay area, PAMF provides integrated, comprehensive health care services to approximately 1 million patients, 30% of whom are Asian, Pacific Islanders, or both. In Hawai’i, KPHI provides comprehensive health care services to approximately 225,000 members throughout the state, over 70% of whom are Asian, Pacific Islanders, or both.

Both sites use electronic health records (EHR), capturing comprehensive information about utilization, diagnoses, medications, and procedures, as well as detailed demographic information. Data is organized in a Virtual Data Warehouse (VDW), which facilitates the efficient sharing and pooling of EHR data using standard variables, variable definitions, and formats while maintaining privacy [17, 18]. The study was conducted with the approval and oversight of Institutional Review Boards at both study sites. The data used in this study are not publicly available, but the corresponding author can be contacted about data requests.

### Study population

Organized by year for the time period 2014—2018, the overall study population included adult patients (ages 18 years and older) at both sites who made at least one ambulatory visit to a primary care provider in the two years prior. Visits to family practice, internal medicine, and obstetric/gynecologic specialists were considered primary care visits.

### Measures

#### Cardiovascular disease

Patients were included in the denominator each year of the study in which they had at least one primary care provider visit in the prior two years. For each year and each patient, the two prior years were searched for the presence of CVD diagnoses. Patients were considered to have prevalent CVD in any given year they had an ICD-9 or ICD-10 diagnosis code indicating CVD (Table S 1) associated with an ambulatory (outpatient), inpatient, or emergency department encounter, or present in the problem list before December 31. Once a patient had an indication of CVD, the condition was considered still present (prevalent) for each subsequent year that the patient remained in the cohort. CVD conditions included coronary heart disease (CHD), stroke (hemorrhagic and ischemic), peripheral vascular disease (PVD), and a summary type, any CVD (Table S 1).

#### Race and ethnicity

Race and ethnicity are terms fraught with misunderstanding and inappropriate biological implications. Race and ethnicity are social constructs that reflect ancestry, geographical origins, culture and cultural identity, economic opportunity, and shared experience, including racism. To the degree that ancestry reflects geographical origins, there can be biological implications, such as the prevalence of sickle-cell trait among populations with origins in areas of high malaria prevalence. However, our goal in this study is to better define the prevalence of CVD in different race/ethnicity groups with limited exploration of possible explanations and no expectation that biological differences will play any significant role.

Race and ethnicity were derived from self-report. Consistent with US Census methods, all available race/ethnicity data were utilized to first define Hispanic and Non-Hispanic populations. Broad race groups were then defined among the non-Hispanic population for inclusion in the study – White, Black or African American, Asian, American Indian/Alaska Native, Native Hawaiian/Other Pacific Islander. For this analysis, all patients were (Non-Hispanic) White, Asian and/or Native Hawaiian/Other Pacific Islander. This study population was then separated into single race and specific multi-race groups with large enough numbers for analysis: White, Asian, Native Hawaiian/Other Pacific Islander, Native Hawaiian/Other Pacific Islander + Asian (PIA), Native Hawaiian/Other Pacific Islander + White (PIW), Asian + White (AW), and Native Hawaiian/Other Pacific Islander + Asian + White (PIAW). Note that the PIA subgroup included individuals who reported *both* Pacific Islander *and* Asian race. The Asian Pacific Islander (API) term refers to all individuals with any Pacific Islander or Asian race singly or in combination, which is how the term is generally used.

Next, within the single race Asian study population, subgroups of single ethnic populations were constructed: Japanese, Chinese, Asian Indian, Korean, and Filipino. Similarly, single ethnic Native Hawaiian and Other Pacific Islander (OPI) subgroups (including all single OPI ethnic groups, the majority of whom had only ‘other Pacific Islander’ listed as their ethnic group) were constructed, resulting in 12 mutually exclusive study population subgroups. The large and diverse combined population of the two study sites enabled us to narrowly focus on the largest disaggregated API populations represented in the combined population. Individuals who additionally reported a racial or ethnic group of “Other” or “Unknown” were excluded from this analysis, as were combinations with other non-API or White race and ethnic groups (i.e., Hispanic, Black or African American, American Indian/Alaska Native). Small single Asian ethnic groups (Vietnamese, Cambodian, etc.) were also excluded due to small sample sizes. As constructed, single Asian ethnic groups included individuals who reported only that single category, thus excluding small mixed-Asian populations. All the Other Pacific Islander single ethnic groups were small and were consequently grouped together into a single category.

### Statistical analysis

Prevalence analyses focused on the most recent five-year period available (2014–2018) to estimate the most current CVD prevalence. Logistic regression models were used to derive prevalence estimates, odds ratios, and 95% confidence intervals for each of the 12 race/ethnicity groups. Models were adjusted for site, gender, and age (as a continuous variable). We use least square means to estimate prevalence at the mean values of all covariates; thus, these estimates represent the prevalence for an average person in our data. CVD prevalence estimates reflect the mean age of the study population, which was 49.6. Odds ratios and their accompanying confidence intervals were calculated for each race/ethnicity group, with ‘Non-Hispanic White’ as the reference group.

## Results

A total of 684,363 unique individuals were included in the study population (Table 1). Nearly 60% of the population was NHW (*N* = 396,359), while 237,107 were Asian, 9,042 were Native Hawaiian/Other Pacific Islander, and 41,855 were API multi-race. The size of subgroup populations ranged from 2,043 (Native Hawaiian) to approximately 78,000 (Asian Indian). Fifty-four percent of the population was female; 43% of the population was between ages 18 and 44 years, 35% were between ages 45 and 64 years, and 22% were age 65 years and older at the time of cohort entry.

**Table 1:** Population characteristics

	**N**	**%**
**All**	684,363	100
**Race**		
**Non-Hispanic White**	396,359	57.9
**Asian Indian**	77,937	11.4
**Chinese**	67,148	9.8
**Filipino**	54,128	7.9
**Japanese**	28,139	4.1
**Korean**	9,755	1.4
**Native Hawaiian Only**	2,043	0.3
**Other Pacific Islander (single ethnic group)**	6,999	1.0
**Pacific Islander + Asian**	11,892	1.7
**Asian + White**	12,637	1.9
**Pacific Islander + White**	7,531	1.1
**Pacific Islander + Asian + White**	9,795	1.4
**Gender**		
**Female**	372,518	54.4
**Male**	311,845	45.6
**Age**		
**18–24**	46,530	6.8
**25–34**	112,685	16.5
**35–44**	135,373	19.8
**45–54**	125,421	18.3
**55–64**	114,130	16.7
**65–74**	83,068	12.1
**75 + **	67,156	9.8
**Age (mean, sd)**	49.61	17.8

**Fig. 1:**
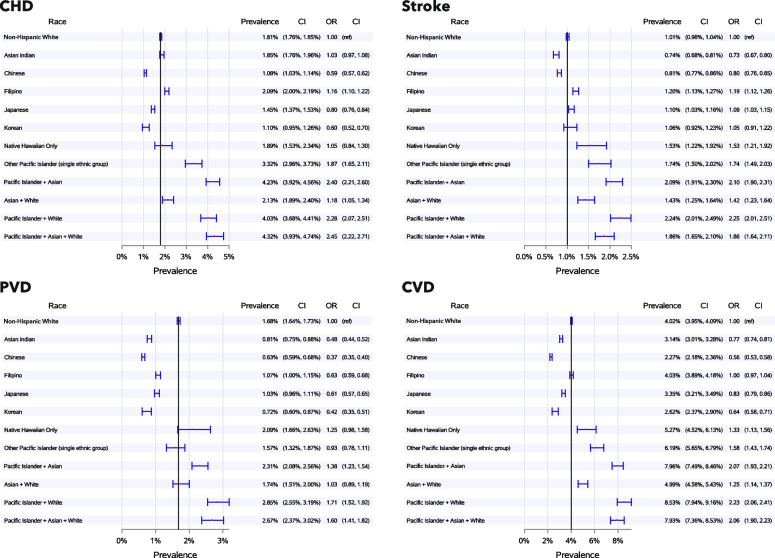
Prevalence of CHD, stroke, PVD, and CVD by race and ethnicity subgroups Prevalence and 95% Confidence Interval (CI) for each race and ethnicity subgroup; Odds Ratio (OR) and 95% CIs are compared to Non-Hispanic White individuals. CHD is Coronary Heart Disease, PVD is Peripheral Vascular Disease, and CVD is the combination of CHD, Stroke, and PVD. Definitions for these diseases are in Table S 1. The vertical line designates prevalence in Non-Hispanic White individuals. Prevalence estimates are adjusted for site, gender, and age as a continuous variable

Figure 1 presents adjusted prevalence estimates and odds ratios with 95% confidence intervals for CHD, stroke, PVD, and overall CVD for each of the 11 racial/ethnic study groups and the NHW comparison group for the most recent 5-year period, 2014–2018.

### Coronary Heart Disease Prevalence (CHD)

Among Non-Hispanic White cohort members (NHW), the prevalence of CHD was 1.81%, while the prevalence of CHD among Chinese, Korean, and Japanese members of the cohort was significantly lower (1.08%, 1.10%, and 1.45%, respectively). Estimates for Other Pacific Islanders and all the multi-race groups were significantly higher than for NHWs, ranging from 2.13% among AWs to 4.32% among PIAW.

### Stroke

The prevalence of stroke among NHW individuals in the cohort was 1.01%. The Asian Indian and Chinese subgroups had significantly lower stroke prevalence than NHWs, with 0.74% and 0.81%, respectively. Estimates for Korean and Japanese members of the cohort were not significantly different from NHWs (1.06% and 1.10%, respectively). All other racial/ethnic groups in the study had significantly higher stroke prevalence than NHWs, ranging from 1.20% among Filipinos to 2.24% among PIWs.

### Peripheral Vascular Disease (PVD)

In comparison to NHWs (1.68%), the prevalence of PVD was significantly lower among all Asian study groups (ranging from 0.63% among Chinese individuals to 1.07% among Filipinos). PVD prevalence among Native Hawaiians, Other Pacific Islanders and the AW multi-race group was comparable to prevalence among NHWs. Higher prevalence was observed among the three other multi-race groups (PIA, PIW, PIAW) in comparison to NHWs.

### Overall CVD prevalence

The estimated prevalence of any CVD among NHWs was 4.02%. For all Asian groups except Filipinos, estimated overall CVD prevalence was significantly lower than that for NHWs, ranging from 2.27% among Chinese individuals to 3.35% among Japanese individuals. Prevalence among Filipinos was not significantly different from NHWs. Observed prevalence among all the Pacific Islander single ethnic and multi-race groups was significantly higher than for NHWs, ranging from 5.27% among Native Hawaiians to 8.53% among PIWs.

As expected, CVD prevalence was lower among women compared to men in the cohort (see Figures S 1 and S 2). Racial and ethnic group prevalence patterns observed in the total population were generally mirrored among both men and women for each of the CVD types.

### CVD patterns among Asians, Pacific Islanders and multi-race API groups

In general, these analyses show a pattern of lower CVD prevalence among Asians and higher prevalence among Pacific Islanders in comparison to NHWs, but there were some notable exceptions. Stroke prevalence among Filipinos was higher than among NHWs, while Japanese and Korean groups were comparable to NHWs. PVD was lower among Asian subgroups in comparison to NHWs, but rates among both Native Hawaiians and Other Pacific Islanders were comparable to NHWs. Among Asians, Filipinos had the highest prevalence of all three CVD conditions and overall CVD, and Chinese people had the lowest prevalence of CHD, PVD and overall CVD. In comparison to Native Hawaiians, Other Pacific Islanders had significantly higher prevalence of CHD; results for stroke and overall CVD were higher than for Native Hawaiians, but not significantly different.

Three of the four multi-race groups included Pacific Islanders (Native Hawaiians and Other Pacific Islanders). For these three groups (PIA, PIW, and PIAW), the prevalence of overall CVD was significantly higher than that for either single-race Native Hawaiians or Other Pacific Islanders, largely due to differences in CHD. The multi-race AW group had significantly higher overall CVD prevalence than both the NHW group and the Asian subgroup with the highest prevalence rate (Filipinos).

## Discussion

### Differences in CVD prevalence among disaggregated API populations

The CASPER study builds on previous work demonstrating the importance of disaggregating API populations for health research, including studies of CVD risk factors, health behaviors, [7, 9, 10, 14, 19–21] and outcomes, including mortality [4, 8, 13, 16, 22–25]. In CASPER, significant differences in CVD prevalence were evident among Asian, Pacific Islander and well-defined multi-race subgroups. The observed prevalence of CHD and PVD was as much as four times higher, and the prevalence of stroke and overall CVD was more than three times higher, in some API subpopulations in comparison to others.

Direct comparison of different studies of CVD among API populations is challenging due to the inclusion of different racial and ethnic populations and classifications. Due to relatively small sample sizes, national-level studies, such as NHIS [7, 26] and NHANES [27, 28], often do not provide estimates for Asian ethnic subgroups. However, CASPER study results are consistent with existing evidence of higher CVD prevalence among Pacific Islanders and lower prevalence among Asians in comparison to NHWs [1, 19, 29].

Importantly, the CASPER study results demonstrate that there are exceptions to this pattern among disaggregated API populations. Prevalence of CHD among Asian Indian men was higher than for NHWs (Figure S 2), consistent with previous literature. CHD and PVD prevalence among Native Hawaiians was not significantly different from NHWs. Among Asian populations, study findings of higher prevalence of all three CVD conditions among Filipinos is consistent with previous reports of higher cardiometabolic risk and poorer health outcomes in this population [4, 7, 13, 15, 19, 22, 30–34]. Among Pacific Islanders, the Other Pacific Islander group had a significantly higher prevalence of CHD in comparison to Native Hawaiians, but similar prevalence of stroke and overall CVD. Results of our study, and other emerging studies demonstrating significant differences in health risks among Pacific Islander groups, support the disaggregation of these populations whenever possible [21, 22, 35, 36].

The finding of higher prevalence for some CVD conditions among some multi-race groups in comparison to their composite single race/ethnic groups was not expected. A previous study comparing hypertension prevalence among people with varying degrees of Hawaiian ancestry reported significantly lower rates associated with higher proportion of Hawaiian ancestry [37]. Overweight and obesity, important risk factors for CVD, were also reported to be most prevalent among those reporting Native Hawaiian ancestry alone, and lowest among those reporting Native Hawaiian ancestry in combination with Asian ancestry [37]. There is a paucity of health information available on multi-race groups in general, and even less for well-defined multi-race groups.

It is possible that individuals in multi-race groups experience more psychosocial stress related to racism, discrimination, and bias than single race minority populations. A Pew Research Center report describes US multi-racial populations as “*at the cutting edge of social and demographic change in the U.S.—young, proud, tolerant and growing at a rate three times as fast as the population as a whole.*” [38] Survey results report both positive attitudes about being multi-cultural as well as negative experiences of racial discrimination. There is evidence that psychosocial stress associated with racial discrimination and bias has a negative impact upon cardiovascular disease risk, including among Pacific Islanders [39, 40]. The finding of higher prevalence of CVD among well-defined multi-racial API populations is novel. Multi-racial populations in the US are becoming increasingly important, and these findings warrant further investigation of the many potential underlying individual, social, and clinical factors that correlate with these differences.

Health disparities among different race/ethnicity groups are often related to differences in demographic, clinical, economic, psychosocial, and other social determinants of health. The inverse association of socioeconomic status (SES) with all phases of the progression of CVD is well established, from the onset of risk factors through mortality due to CVD [41–45]. Immigration status and acculturation processes may also impact CVD risk among Asian and Pacific Islander populations [46]. Control of blood pressure, dyslipidemia, and diabetes are critical to primary and secondary prevention of CVD. These risk factors may develop at earlier ages among some population groups, be identified at different stages, and may be treated with different intensity. Patients’ experience of health care is also affected by perceived discrimination, resulting distrust in the provider, and, potentially, racial concordance with providers [47–51]. Understanding these underlying and potentially modifiable factors contributing to the health disparities is critical to designing and implementing effective intervention strategies to reduce CVD mortality and morbidity among these populations. The CASPER study includes data on many potential demographic, clinical, and psychosocial factors that will be explored in future analyses.

### Implications for the classification of Asians, Pacific Islanders, and multi-race people in research

Results of the CASPER study further support the need to disaggregate Asian and Pacific Islander populations to study CVD and other health conditions. Clearly, researchers need to recognize that the Asian and Pacific Islander groups are not homogeneous; this is also true of other convenient groupings, such as Hispanic and Black.

Classification of multi-race individuals for research is particularly challenging. Efforts in the past have reduced multi-race populations to a single category by assigning them to either the largest or smallest group they belong to, asking which one race the person most identifies with, or grouping all multi-race individuals into a single category (e.g., two or more races) [52, 53]. They are also often excluded from research entirely. Some additional considerations include the fluidity of self-identification among multi-racial people, which can vary in different social contexts and change over time [54]. Similarly, we cannot assume that multi-race individuals would necessarily identify more with one race they belong to than another [38]. Data collection of self-reported race and ethnicity has shifted to accommodate this demographic trend, enabling people to report multiple races and ethnic groups. For health researchers, this results in multiple discrete combinations, many with small population sizes, some of which are difficult to interpret, such as multiple race combinations that include ‘other’ or ‘unknown.’ There is no perfect approach, and combining or excluding such groups becomes unavoidable. To the extent possible, studies should use a classification scheme that best fits the study population, capturing small groups and well-defined multi-race groups when feasible. With such rapid population growth, it becomes increasingly important to consider potential differences among multi-racial groups and to capture study results for the most common multi-racial combinations when possible.

### Study limitations and strengths

The CASPER study makes an important contribution to knowledge about CVD among disaggregated single race and multi-racial API populations. With recent five-year EHR data for a total population of more than 684,000 people, including more than 237,000 Asians, 9,000 Pacific Islanders, and nearly 42,000 multi-race API people, it is among the largest studies of its kind. It was conducted in two US geographic regions in the states with the largest API populations in the US, and included all adults aged 18 years and older enrolled in two health plans. Importantly, the study included patients receiving care from two different health care systems and CVD was defined by clinical evidence contained in EHR data.

The CASPER study also has some limitations. Although California and Hawai’i have the largest populations of Asians and Pacific Islanders in the country, results may not be generalizable to other US API populations. Different Asian populations are clustered in different parts of the country and individuals of the same group may have different experiences related to population diversity in the region. The population subgroups in this study were not uniformly distributed across the two sites; South Asians are more common in the California sample and Pacific Islanders in Hawai’i. This study was limited to people with health insurance, and likely underrepresents recent immigrants and people with lower incomes. Individuals from these excluded groups may have an even greater burden of CVD compared to the CASPER population. However, individuals and subpopulations in the CASPER study may have experienced different barriers to health care, or differences in ‘realized’ access, despite having insurance. The study did not include Hispanic or Black populations. In addition, this analysis did not include individual level socioeconomic measures, an important consideration for future studies to determine differences in CVD prevalence by race. Finally, the study was limited to two health care systems (one integrated health care organization and one multi-payer system), which could limit generalizability of results to populations served by other health care systems.

### Conclusions

Study findings provide evidence of significant differences in overall CVD, as well as CHD, stroke, and PVD, among the 11 study API groups and the NHW comparison group. In addition to elevated risk among Filipinos, Native Hawaiians, and Other Pacific Islanders, study results identified particularly elevated risk among multi-race API groups. Differences in disease risk are likely to be mirrored in other cardio-metabolic conditions, supporting further application of the disaggregation of API groups in health research. CASPER data will continue to be explored to identify underlying and potentially modifiable demographic, clinical, and psychosocial factors that may result in observed racial and ethnic differences in CVD.

## Supplementary Information

**Additional file 1: Table S1.** Definitions of Study Cardiovascular Disease (CVD) Conditions According to International Classification of Disease (ICD) Codes.**Figure S1.** Prevalence of CHD, stroke, PVD, and CVD in women by race and ethnicity subgroups. **Figure S2.** Prevalence of CHD, stroke, PVD, and CVD in men by race and ethnicity subgroups.

## Data Availability

Not publicly available.

## Race

API: Asian and Pacific Islander (all)
AW: Asian + White
NHW: Non-Hispanic White
PIA: Pacific Islander + Asian
PIAW: Pacific Islander + Asian + White
PIW: Pacific Islander + White

## Cardiovascular disease

CHD: Coronary heart disease
CVD: Cardiovascular disease
PVD: Peripheral vascular disease
