# Supplementary material for: Prevalence of cardiovascular disease among Asian, Pacific Islander and multi-race populations in Hawai’i and California
Source: BMC Public Health. 2023 May 15;23:885. doi: 10.1186/s12889-023-15795-5 (PMC10184427; doi:10.1186/s12889-023-15795-5)
Supplement: Supplementary file 1 — Additional file 1: Table S1. Definitions of Study Cardiovascular Disease (CVD) Conditions According to International Classification of Disease (ICD) Codes.Figure S1. Prevalence of CHD, stroke, PVD, and CVD in women by race and ethnicity subgroups. Figure S2. Prevalence of CHD, stroke, PVD, and CVD in men by race and ethnicity subgroups. [file 12889_2023_15795_MOESM1_ESM.docx]

**Table S1.** Definitions of Study Cardiovascular Disease (CVD) Conditions According to International Classification of Disease (ICD) Codes

| **Description** | **CASPER Prevalence**  **Cases ICD-9, *ICD-10*** |
| --- | --- |
| **CHD – Myocardial Infarction** | 410, 412  *I21, I22, I25.2* |
| **CHD – Other ischemic heart disease** | 411, 413, 414  *I20, I24, I25 (except I25.2)* |
| **Hemorrhagic stroke** | 430-432, 438, V12.54  *I60, I61, I62, I69.9, Z86.73* |
| **Ischemic stroke** | 433, 434  *I63, I65, I66* |
| **PVD – Peripheral arterial disease** | 440.2, 440.3, 443.9  *I70.2x-170.7x, I73.9* |
| **PVD – Venous disease** | 415, 451, 453  *I26, I80, I82, T81.718A, T82.818A* |

Each diagnosis had to appear in at least one clinical encounter, excluding email, telephone, lab-only, radiology-only, non-acute institutional stay, and “other,” or in the problem list diagnoses before or during the denominator year


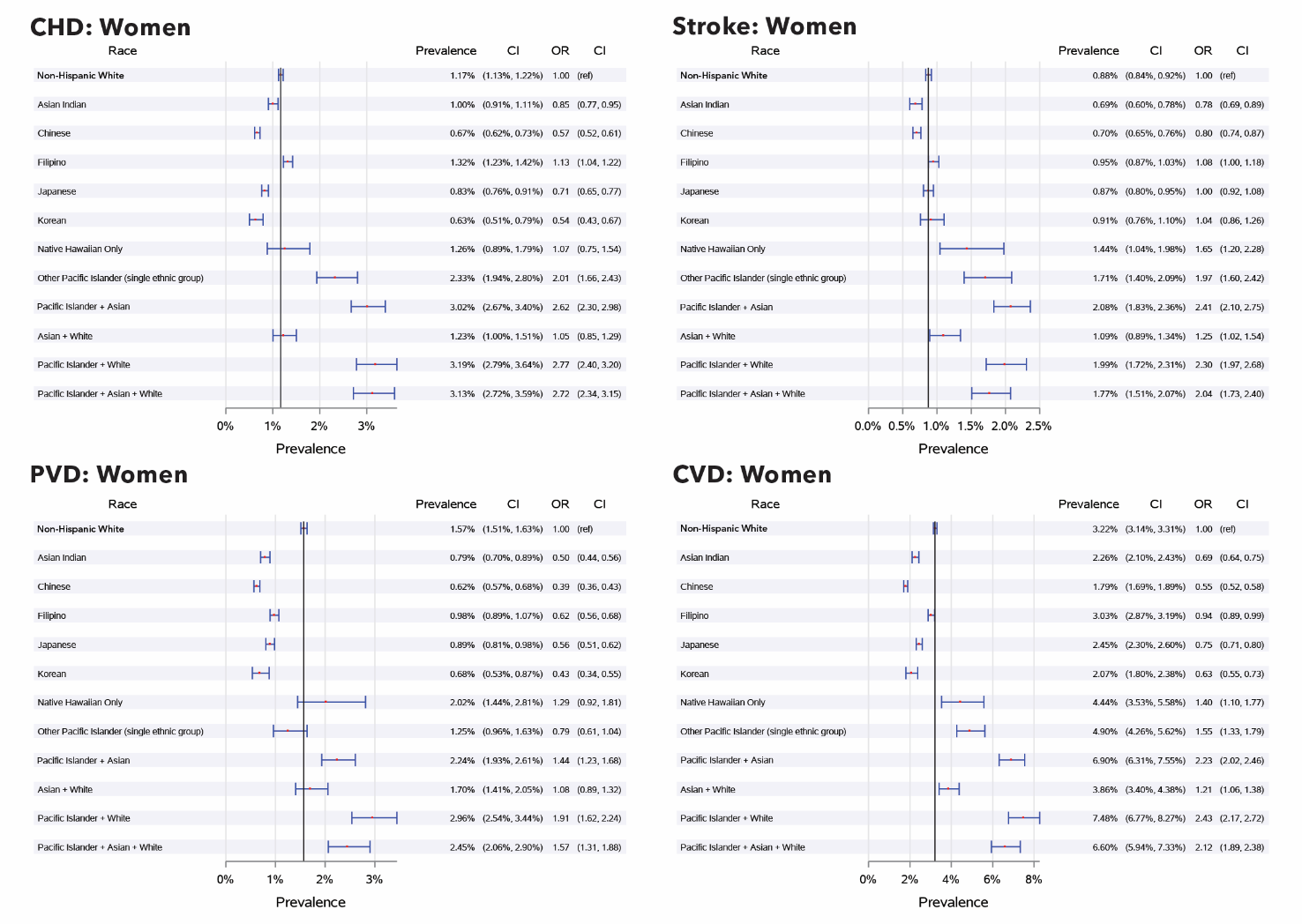


**Figure S1. Prevalence of CHD, stroke, PVD, and CVD in women by race and ethnicity subgroups**.

Prevalence, 95% Confidence Interval (CI), Odds Ratio (OR) compared to Non-Hispanic White individuals, and 95% CI for the OR. CHD is Coronary Heart Disease, PVD is Peripheral Vascular Disease, and CVD is the combination of CHD, Stroke, and PVD. Definitions for these diseases are in Table S1. The vertical line designates the prevalence in whites. Prevalence estimates are adjusted for site and age as a continuous variable.


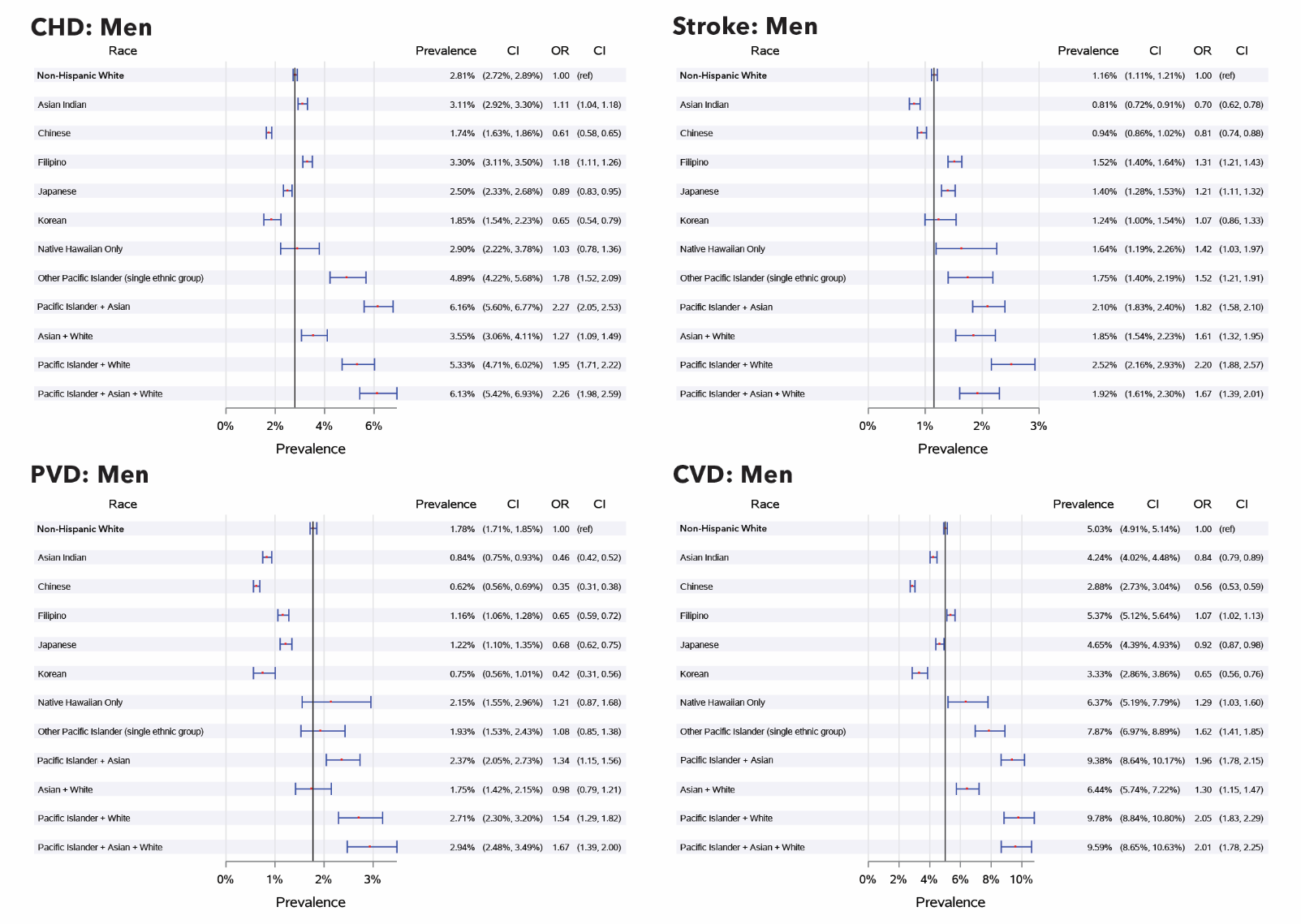


**Figure S2. Prevalence of CHD, stroke, PVD, and CVD in men by race and ethnicity subgroups**.

Prevalence, 95% Confidence Interval (CI), Odds Ratio (OR) compared to Non-Hispanic White individuals, and 95% CI for the OR. CHD is Coronary Heart Disease, PVD is Peripheral Vascular Disease, and CVD is the combination of CHD, Stroke, and PVD. Definitions for these diseases are in Table S1. The vertical line designates the prevalence in whites. Prevalence estimates are adjusted for site and age as a continuous variable
